# Thermalization of Mesh Reinforced Ultra-Thin Al-Coated Plastic Films: A Parametric Study Applied to the Athena X-IFU Instrument

**DOI:** 10.3390/s24072360

**Published:** 2024-04-08

**Authors:** Nicola Montinaro, Luisa Sciortino, Fabio D’Anca, Ugo Lo Cicero, Enrico Bozzo, Stéphane Paltani, Michela Todaro, Marco Barbera

**Affiliations:** 1 Dipartimento di Ingegneria, Università Degli Studi di Palermo, Viale delle Scienze, Edificio 8, 90128 Palermo, Italy; 2 Istituto Nazionale di Astrofisica (INAF), Osservatorio Astronomico di Palermo, Piazza del Parlamento 1, 90134 Palermo, Italy; 3 Département D’astronomie, Faculté des Sciences, Université de Genève, Chemin d’Ecogia 16, 1290 Versoix, Switzerland; 4 Dipartimento di Fisica e Chimica-Emilio Segrè, Università Degli Studi di Palermo, Via Archirafi 36, 90123 Palermo, Italy

**Keywords:** thermalization, FEA, heat transfer analysis, detector, X-ray filter, Athena, X-IFU

## Abstract

The X-ray Integral Field Unit (X-IFU) is one of the two focal plane detectors of Athena, a large-class high energy astrophysics space mission approved by ESA in the Cosmic Vision 2015–2025 Science Program. The X-IFU consists of a large array of transition edge sensor micro-calorimeters that operate at ~100 mK inside a sophisticated cryostat. To prevent molecular contamination and to minimize photon shot noise on the sensitive X-IFU cryogenic detector array, a set of thermal filters (THFs) operating at different temperatures are needed. Since contamination already occurs below 300 K, the outer and more exposed THF must be kept at a higher temperature. To meet the low energy effective area requirements, the THFs are to be made of a thin polyimide film (45 nm) coated in aluminum (30 nm) and supported by a metallic mesh. Due to the small thickness and the low thermal conductance of the material, the membranes are prone to developing a radial temperature gradient due to radiative coupling with the environment. Considering the fragility of the membrane and the high reflectivity in IR energy domain, temperature measurements are difficult. In this work, a parametric numerical study is performed to retrieve the radial temperature profile of the larger and outer THF of the Athena X-IFU using a Finite Element Model approach. The effects on the radial temperature profile of different design parameters and boundary conditions are considered: (i) the mesh design and material, (ii) the plating material, (iii) the addition of a thick Y-cross applied over the mesh, (iv) an active heating heat flux injected on the center and (v) a Joule heating of the mesh. The outcomes of this study have guided the choice of the baseline strategy for the heating of the Athena X-IFU THFs, fulfilling the stringent thermal specifications of the instrument.

## 1. Introduction

The Advanced Telescope for High-Energy Astrophysics (Athena) [1] is a large-class astrophysics space mission selected by ESA to address the hot and energetic universe science theme [2], expected to be flown in an L1 orbit in the mid-2030s. Athena will be equipped with a 12 m focal length grazing incidence X-ray telescope based on silicon pore optics technology [3], capable of providing a >1.0 m^2^ effective area at 1 keV with an angular resolution full width at half maximum (FWHM) better than 10″ over a large field of view (>40′ diameter). The telescope will be mounted on a moveable platform to allow both focus adjustment and tilting to point the X-ray beam on one of the two focal plane detectors: the X-ray Integral Field Unit (X-IFU) [4], a micro-calorimeter array, and the large Wide Field Imager (WFI) [5], an array of depleted field effect transistors (DepFET).

The mission has recently undergone a redesign phase to reduce cost and complexity, which was successfully completed in November 2023. The Science Policy Committee (SPC) of ESA has approved the new Athena concept with a clear statement that it remains a science flagship mission, and a new delta phase A was started in Q1 2024.

The X-IFU will consist of an array of Transition-Edge Sensor microcalorimeters sensitive in the 0.2–12 keV energy range, featuring an energy resolution of <3.0 eV at 7 keV.

The X-IFU detectors will be operated at a temperature of <100 mK, requiring a multi-stage cryostat to guarantee the requested temperature. The attenuation of the infrared radiative load is mandatory to minimize the energy resolution degradation due to photon shot noise [6]. For this reason, a set of five thermal filters (THFs) will be located at some cryostat stages creating an open path to the aperture cylinder (ApC) [7,8], allowing the X-ray photons to reach the detector. The definition of the number of filters and their thickness has been identified by performing an optimization analysis to obtain the best efficiency at the price of an energy resolution degradation due to photon shot noise within the requirements [9].

THFs must be highly transparent in the soft X-ray energy range while reflecting infrared radiation. Furthermore, they shall protect the detector from molecular contamination and low-energy particles.

In the case of instruments onboard previous X-ray missions, such as the Advanced CCD Imaging Spectrometer (ACIS) instrument onboard the Chandra mission, the molecular contamination of the filters was identified as one of the crucial causes of the instrument progressive loss of sensitivity at low energy since the beginning of the scientific operation [10].

The main atoms of the contaminants were identified as carbon, oxygen, and fluorine. The most likely sources of contaminants are now understood to be carbonaceous compounds, fluorocarbon compounds, and water [11]. The heritage gained with the ACIS instrument over the past 20 years, combined with the studies and modeling conducted so far to evaluate the possibility of baking filters devoted to space applications to clean their surfaces, are a good starting point to understand to what extent the build-up of contaminants can be an issue for the X-IFU filters.

A careful investigation related to molecular contamination effects and the associated need for a thermal control of a few filters in the stack shielding a microcalorimeter in space has been performed for the design and development of the Soft X-ray Spectrometer (SXS) onboard the Astro-H (Hitomi) [12]. The same approach has been replicated for the SXS, named Resolve, onboard the X-Ray Imaging and Spectroscopy Mission (XRISM) [13]. For the X-IFU microcalorimeter, a detailed mechanical and thermal design of the Aperture Cylinder (ApC) with a similar approach has been performed to minimize the risk of contamination on the inner filters and detector [8]. The temperature control for contamination issues has been implemented on the outer filter via heaters placed on the filter carrier, while a detailed thermal analysis and optimization design of the outer filter to reach the required temperature profile is necessary for this study.

**Figure 1:**
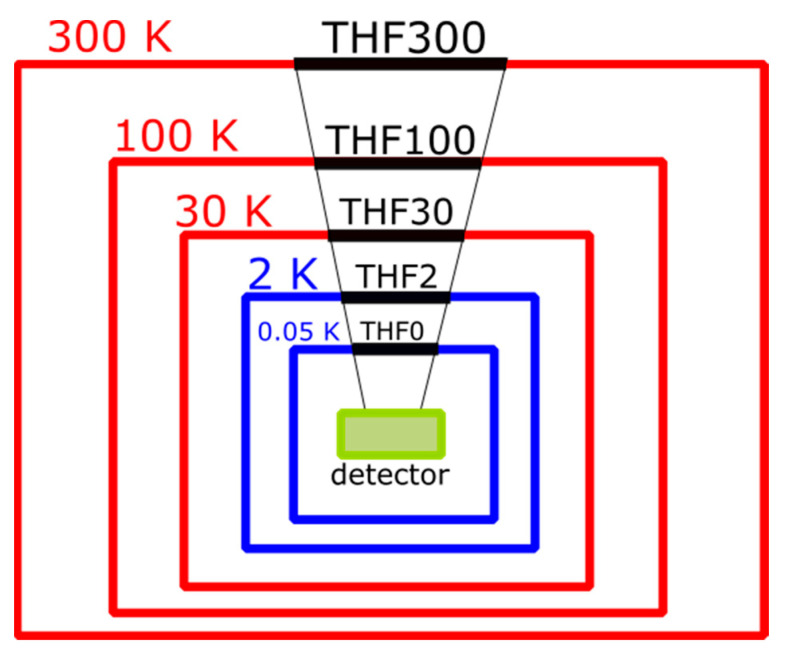
Schematic mounting of the thermal filters in the X-IFU cryostat. The THF300, THF100, and THF30 are mounted on the cryostat shields (red boxes) kept at 300 K, 100 K, and 30 K, respectively. The THF2 and the THF0 are mounted on the Focal Plane Assembly stages (blue boxes) at 2 K and 50 mK, respectively.

Considering that the X-IFU assembly design has five filters, the contaminant accumulation could severely affect the sensitivity at low energies if several filters are contaminated. In the current mission baseline, the five filters of the stack are mounted and thermally anchored to five different cryostat shields operating at nominal temperatures of 300 K, 100 K, 30 K, 2 K, and 0.05 K. Figure 1 provides a schematic cross-section of the dewar.

Each filter is named by the nominal temperature of the shield on which it is mounted (e.g., THF2 at 2 K), for simplicity, the coldest one operating at 0.05 K is named THF0. The first barrier from external contaminants is the largest THF300, installed on the outer vessel of the cryostat. The outer filter, which is potentially more exposed to contamination, should be kept at a higher temperature than the 320 K environment to minimize molecular contamination that can already occur at this temperature. The THF100 and THF30 are also mounted on the ApC, while the last two filters, the THF2 and THF0 (operating at 50 mK), are directly mounted on the Focal Plane Assembly (FPA), the inner core of the instrument.

A description of the thermal environment of the X-IFU detector and, in particular, the temperature stages where filters are mounted is provided in the report [14] prepared by the X-IFU instrument consortium as part of the data package delivered to ESA in March 2022 for the Instrument–System Requirement Review, and also described in the paper by Barret et al., 2023 [4].

The baseline design of the THFs achieved so far relies on the expertise matured through previous X-ray missions, such as the Chandra and XMM-Newton missions. In these cases, the filters survived the mechanical stresses of the launch and are still working with no significant chemical and physical degradation after over 20 years in space [15]. According to the current design, all THFs will be made of a thin film of polyimide (45 nm) coated with aluminum (30 nm) to meet the low energy effective area requirements [16]. As the thermal conductance of these thin films is very poor, their large membranes are prone to developing a temperature gradient between the outer and the inner part. A hexagonal metallic mesh will be adopted to mechanically support these thin membranes. Moreover, to alleviate the temperature gradient, the supporting mesh can be appropriately designed to improve the heat conductivity of the filters [17]. Considering the high aspect ratio of the THF membrane and, consequently, their fragility, measurements of their temperature gradient via in situ probes (e.g., thermocouples) are not possible. An alternative approach could be IR-thermography, which would allow us to acquire fast, contactless, and full-frame acquisition of the thermal gradient. However, the successful application of this technique is hampered by the negligible emissivity of the membrane aluminized surface in the IR energy domain. 

In this scenario, a parametric numerical study thus became the only viable solution to probe different filter configuration trade-offs. To the best of our knowledge, the available literature in the field of thermalization strategies for nanometric structures in the stringent boundary conditions applicable to X-ray satellite instrumentation (Cryogenic temperature, risk mitigation, etc., …) is still scarce.

In this work, a parametric numerical study is performed on different technical solutions for the Athena X-IFU thermal filter THF300 heating with the goal of minimizing molecular contamination during the Athena mission lifetime. The thermal model retrieves the radial temperature profile on the THF300 filter and establishes the necessary conditions to keep the whole filter area above the temperature of 320 K. The outcomes of this study have guided the choice of the baseline heating strategy compliant with the stringent thermal specifications of the X-IFU instrument, the selection of the filter mesh and plating materials, and the required operating temperature of the filter carrier mounted on the 300 K shield. In the parametric numerical study, the effects of different boundary conditions on the radial temperature profile have been considered, namely: (i) the mesh design and material, (ii) the plating material, (iii) the addition of a thick Y-cross applied over the mesh, (iv) an active conductive heating injected on the center, and (v) a distributed Joule heating of the mesh.

The research work reported here is part of the activities that have led to the successful completion of the data package submitted to ESA in Q1 2022 for the Athena X-IFU Instrument System Requirement Review. As of May 2022, ESA has decided to not bring Athena into the Mission Adoption Review, requesting a significant reformulation of the mission to reduce costs. The exercise was completed in November 2023, with a formal approval of the reformulated Athena mission by the Astronomy Working Group (AWG) and Space Policy Committee (SPC) and a clear statement that the reformulated Athena mission still remains an ESA flagship mission. The kick-off of a new delta phase A/B1 for Athena is scheduled in Q1 2024. Though the new X-IFU will require a different filter configuration, the significant work performed before the reformulation exercise constitutes a significant heritage of design and technology assessments, on which we will build upon the development activities of Athena in the new configuration.

## 2. Baseline Design and Cases Studied

As reported before, previous X-ray experiments, such as the ACIS instrument [10], suggest the possibility of controlling the filter temperature to prevent the deposition of contaminants. To minimize contamination, the larger and outer filter (THF300) should be maintained at a temperature higher than the surrounding environment (300 K). In this study, a target temperature of 320 K was used.

**Figure 2:**
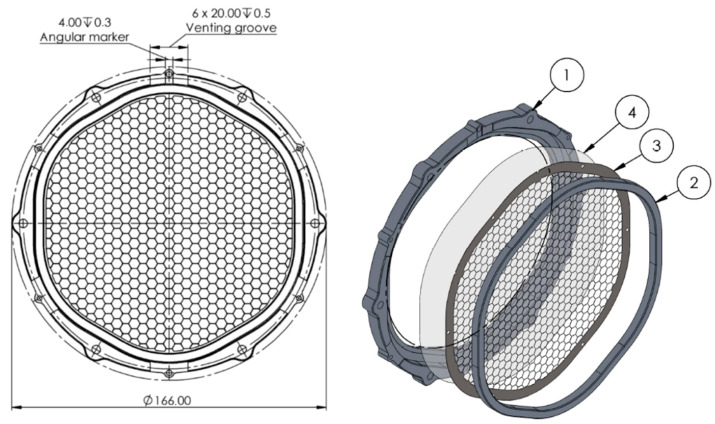
On the left panel is the front view of the THF300 filter assembly; on the right panel an exploded view of the four parts of the filter: (1) outer frame, (2) inner frame, (3) mesh, (4) aluminized polyimide membrane.

The baseline design of the THF300 comprises an outer frame, an inner frame, and a metallic mesh reinforcing the thin aluminized polyimide (PI) film (see Figure 2). The clear aperture diameter of the hexagonal metal frame with rounded corners is 132 mm. The PI film is 45 nm thick and is coated with 30 nm of aluminum. The reinforcing mesh in its baseline design has a hexagonal pattern with a pitch of 5.3 mm and a mesh arm dimension height × width (h × w) of 80 µm × 40 µm.

Different design variations to thermalize the THF300 have been investigated by numerical simulations. The proposed solutions can be summarized into two different classes: passive conduction or active heating.

The following heating options are considered for the passive conduction approach:

1. Raising the temperature of the external frame and heating the filter by conduction through the mesh;
2. Raising the temperature of the external frame and heating the filter by conduction through the mesh and an additional thick Y-shaped aluminum structure applied above the mesh.

The following heating options are considered for the active heating approach:

3. Injecting a heat flux at the center of the filter and heating the filter by conduction through the mesh;
4. Running an electrical current through the mesh from the center to the external frame and heating the filter via a distributed Joule heating.

In the first set of four cases, the temperature of the external frame is fixed at 300 K, thus evaluating the influence of each parameter on the THF300 thermalization. The first two cases are related to passive conditions, while for the third and fourth cases, active heating conditions were considered as an amount of energy injected to promote thermalization.

For the last four cases (from the fifth to the eighth), the external frame heating is used to boost the thermalization to reach at least 320 K throughout the filter. A brief description of each of the eight cases is presented below.

**Figure 3:**
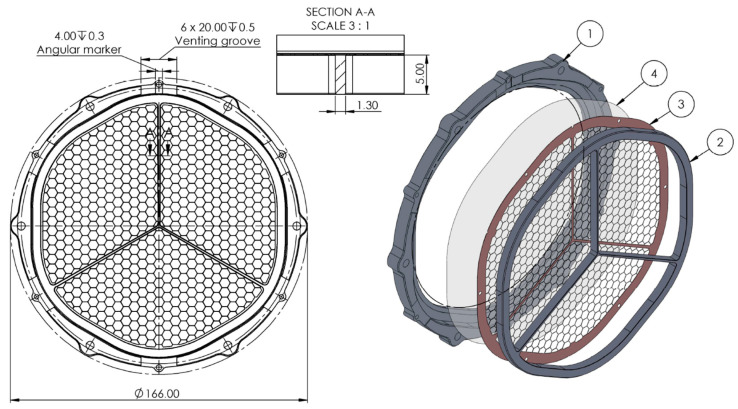
On the left: front view of the THF300 assembly with the Y-cross added to the inner frame. On the right: exploded view of the four parts of the THF300 filter: (1) outer frame, (2) Y-cross, (3) mesh, (4) aluminized polyimide membrane.

The first case evaluates the effect of the mesh and plating material. In the second and sixth cases, a Y-cross structure is added above the mesh (Figure 3) to improve the thermal conductance of the filter from the external frame towards the center.

As can be seen from Figure 3, the cross-section of the Y-cross arm is several orders of magnitude thicker (5 mm × 1.3 mm) than the mesh arm, and thus the heat flux is increased. The main cons of this design are the increased blocking factor (BF up to 6%) for the X-rays that will instead be revealed by the TES detectors, and the additional shadowing effects due to the non-uniformity of the filter which can affect the images produced by the instrument once they are projected onto the FPA.

In the third and seventh cases, active heating is implemented by adding a thin film resistor at the mesh center activated by running a current through it. In this scenario, it is possible to control the injected heat, and thus the resulting temperature of the filter meets the requirement by modulating the current. The active heating can be set in continuous mode or in a specific time frame for decontamination only. To install the resistor on the center of the filter, a central hexagonal cell of the mesh should be closed adding a localized mass and slightly increasing the BF. In addition, to run a current through the resistor, a wire should be connected to the resistor. This would increase the risk of failures during launch due to vibrations.

In the fourth and eighth cases, a Joule heating solution is proposed. In this option, instead of placing a resistor at the central cell and locally heating the mesh, a wire is connected at the mesh center running a current through the mesh towards the external grounded frame. In this heating strategy, the mesh is heated thanks to the effect of the Joule, smoothing out the thermal gradients. In this latter case, the mesh material is a critical variable since the electrical resistance plays a key role in the generation of the required heat. As in the previous case, the main risk of failure in this approach is due to the wire connected to the center and the need for a blind central cell which puts a concentrated mass in the center, which may locally stress the mesh and membrane during vibrations.

To explore possible solutions to improve the conductive heating of the THF300 filter, for each of the eight case studies listed in Table 1, four alternative mesh designs, in addition to the baseline, are proposed as illustrated in Table 2. The changes involve the mesh material, mesh thickness, plating materials, as well as the general dimensions of the mesh (height, width, and plating thickness). In the following, we identify the different model configurations investigated for each heating case study with the letters A, B, C, D, E with model A being the baseline.

**Figure 4:**
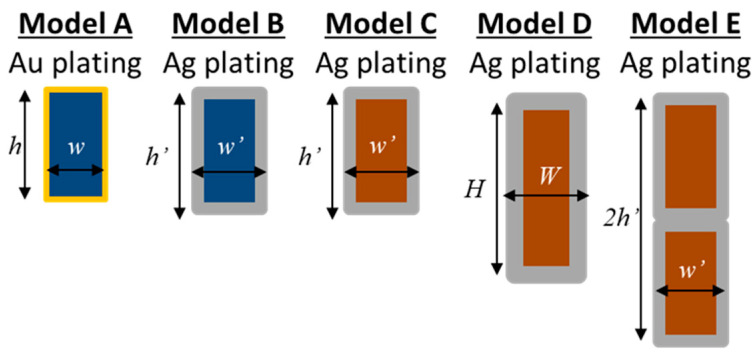
Schematic representation of the cross-sections of the THF300 mesh arm for the five proposed models: model A represents the baseline with stainless steel (blue) plated with gold (yellow), model B has the same mesh material with a thicker silver plating (grey), model C has the BeCu mesh (brown) with silver plating, model D is the optimized BeCu mesh design, described in the next picture with the thickest silver plating, and model E is the double BeCu mesh with silver plating. The dimensions h, h’, H and w, w’, W, are 90, 100, 110 µm and 50, 60, 70 µm, respectively.

A schematic representation of the mesh bar cross section of the five modeled solutions is schematically represented in Figure 4, where the total mesh arm height and width are calculated including the metal plating.

**Figure 5:**
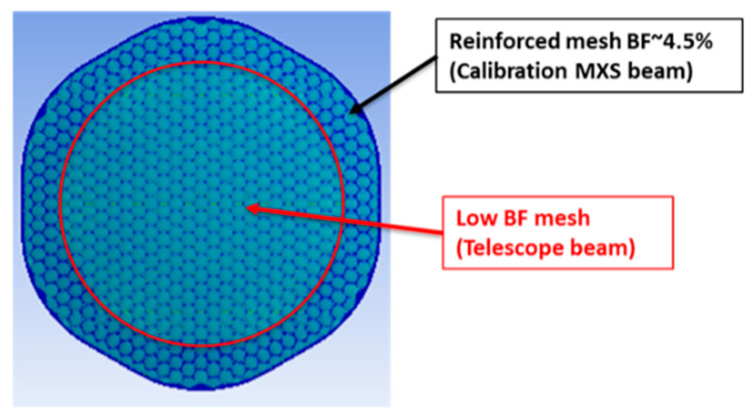
Graphical representation of the optimized mesh design used on the model D. The mesh has wider mesh bars in the outer ring illuminated only by the calibration modulated X-ray sources.

A hexagonal SS AISI304 mesh coated with 5 µm of gold is the baseline design and is identified as model A. In model B, the plating material is silver with a 10 µm thickness while model C differs from model B for the adoption of a different mesh material (the BeCu C17200 alloy [18]). The increased thickness of the plating for model B slightly affects the BF while enhancing thermal conductivity. Since the density of the Ag is almost half of the Au, the total weight of the plated mesh between model A and B is almost equal. In model C, the mesh material is substituted by the BeCu alloy which enhances the poor thermal conductivity of the SS and preserves its strength (BeCu C17200 alloy has comparable ultimate and yield strength [19]). In model D, the thermal conductivity of the mesh is enhanced using a thicker layer of Ag (15 µm) over a BeCu alloy C17510 [20] (which doubles the thermal conductivity of the C17200 with a slight decrease of the ultimate and yield strength). Depositing a large thickness of Ag is a technological challenge due to the high aspect ratio concerning mesh bar width (40 μm nominal). Filter breadboards have been manufactured with BeCu meshes with a nominal Ag plating thickness of 15 μm, and technological difficulties have been encountered in obtaining flat and good surface finishing meshes. Moreover, the mesh of model D has an optimized geometry with increased cross-sectional arms area out of the telescope beam cone (see Figure 5). This design allows for increasing the heat flux towards the filter center without affecting the BF of the X-IFU instrument. The slightly higher BF of the model D (from 2.75% to 3.1%), with respect to models A, B and C, is attributed only to the thicker Ag plating (from 10 μm to 15 μm).

## 3. Thermal Modeling

### 3.1. Numerical Simulation Geometry and Assumptions

**Figure 6:**
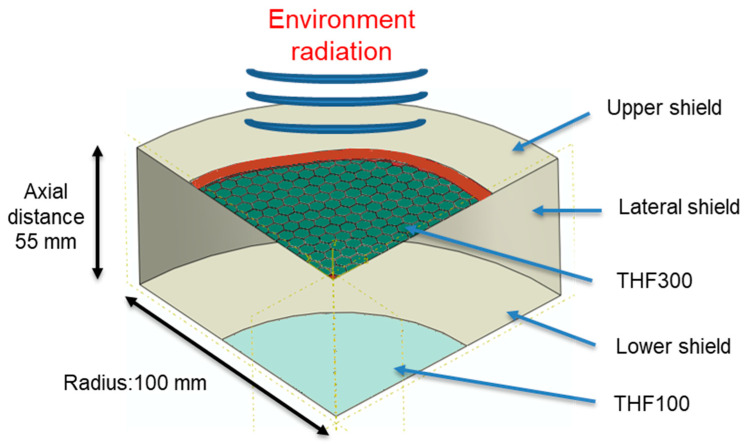
A 3D representation of the quarter of FEM static heat-transfer simulation with its boundary conditions (symmetries of the sector, temperatures and emissivity).

To retrieve the radial temperature profile of the THF300 when it is mounted on its cryostat shield, a steady-state heat transfer finite element model (FEM) has been used. Since the X-IFU instrument is planned to operate in a vacuum, the only thermal interaction between the model parts is through conduction and radiative heat transfer. Considering the inner frame hexagonal shape with rounded corners and the hexagonal mesh pattern, to preserve accuracy, a 3D model was adopted instead of an axisymmetric one. To save computational time, only a quarter of the filter was considered (see Figure 6). Since the commercial FEM softwares does not take into account the transmitted light, only the emitted and reflected components are included in this simulation, neglecting the transmitted component. This assumption is an acceptable approximation since the transmission of a 30 nm thick Al coating in the IR at 10 μm (peak wavelength of a 300 K blackbody) is lower than 0.001, thus the main components driving the thermal equilibrium are emission and reflection [19]. The geometry of the whole model is taken from the dewar assembly of the X-IFU instrument [7]. As shown in Figure 6, the THF300 is loaded with the environmental radiation coming from the warmer cryostat section on the upper surface (300 K). The bottom THF300 surface is instead exposed to a cylindrical closed cavity delimited from the lateral and upper/lower shields and from the THF100 filter which is modeled as circular for simplicity. Each surface of the model has its specific emissivity and temperature.

**Figure 7:**
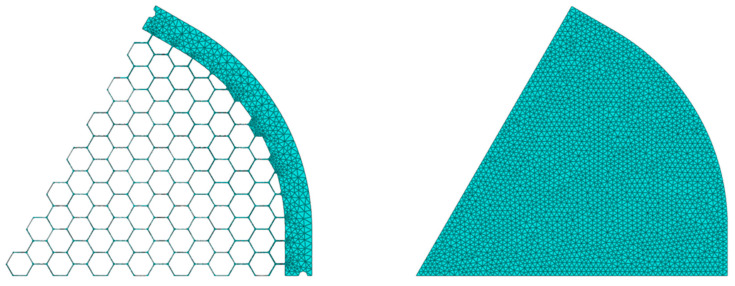
Discretization of the hexagonal mesh (**left panel**) and discretization of the composite membrane (**right panel**).

The upper shield is set at a fixed temperature of 300 K while the lateral/lower shields and the THF100 filter are set at 100 K [7]. The surface emissivity of the THFs is set to 0.02 while all the shields are set to 0.8 [21,22]. The cavity has been approximated as straight cylindrical, while the THF300 was modeled according to the case study under investigation. The high aspect ratio of the filter parts (membrane and mesh) allows us to save computational time without reducing accuracy, discretizing the membrane/mesh with planar shell element instead of volumetric brick element. The thin membrane structure, which we recur to be composed of two layers, 45 nm of PI and 30 nm of Al, is modeled as a composite structure with independent and in perfect contact layers. The membrane and mesh parts are bonded assuming a perfect contact interaction between element nodes. A preliminary convergence study has been performed to ensure robust results with the adoption of a sufficient discretization rate. A 4-node heat transfer quadrilateral shell has been used to discretize the mesh with 1.5 mm size, while a 3-node triangular shell with 1 mm size mesh is used to discretize the composite membrane (see Figure 7).

### 3.2. Material Properties

The thermal conductivities—k—of each material are set considering the temperature-dependent law. The thermal conductivity trends as a function of the temperature were taken from gold [23], polyimide [24], aluminum 5N [25], silver 5N [23,26], BeCu C17200 [18], and C17510 [27]. Table 3 reports the thermal conductivity of the chosen material at the nominal temperature of 300 K.

To simplify the simulation of the plated mesh, the rule of mixture was used, weighting the base material with the plating, each one with its respective cross sections, ergo, the thermal conductivity of the mesh—*k_mesh_*—is computed as follows:

$$k_{mesh}\left(T\right)=k_{core}\left(T\right)A_{core}+k_{plating}\left(T\right)A_{plating}$$

where *k_core_* and *k_plating_* are the thermal conductivities of the mesh and the plating materials, while *A_core_* and *A_plating_* are the fractional cross-section areas of mesh and plating, respectively.

The possible source of errors of the proposed model can be attributed to:

- The geometries simplification on the cavity and on the THF100;
- The THF100 boundary conditions (The THF100 is kept at fixed temperature);
- The cross-section of the mesh arm is considered nominal (manufacturing processes could affect the real geometry;
- The perfect contact between the mesh/film and external frame/carrier;
- Uncertainty of the thermal conductivity of the materials (the purity of the metals can affect the thermal conductivity; see Al 5N [25]).

### 3.3. Comparison with an Analytical Model

To validate the FEM approach, a preliminary comparison with a simplified analytical model has been performed for the baseline A–SS Au filter. In the analytical formulation a uniform circular membrane with 67.5 mm of radius (the circumference inscribed the hexagonal shaped THF300 frame), made of an equivalent material, is kept at 300 K on its external edge while immersed in a radiative environment on both sides (300 K above and 100 K below); the emissivity of both surfaces is set to 0.02. The equivalent material for the baseline comparison is obtained by combining the polyimide, aluminum, SS and gold plating with the rule of mixture formula. To compute the apparent thermal conductivity of the mesh/plating (SS/Au) the blocking factor is adopted for spreading the total volume over the entire membrane surface, thus deriving the apparent thickness. The analytical solution for a circular film is then presented in [29]:

$$\frac{\partial^{2}T}{\partial r^{2}}+\frac{1}{r}\frac{\partial T}{\partial r}+\frac{\varepsilon \sigma }{k_{eq}t_{eq}}\left(T^{4}-T_{UP}^{4}\right)+\frac{\varepsilon \sigma }{k_{eq}t_{eq}}\left(T^{4}-T_{DOWN}^{4}\right)=0$$

with *ε*, *k_eq_* and *t_eq_*, the emissivity on both faces of the filter and the thermal conductivity and thicknesses of the equivalent membrane, respectively.

From a macroscopic point of view, the filter is, thus, assumed to be isotropic. Using an equivalent thermal conductivity for the mesh assumes a negligible temperature gradient within each cell of the hexagonal mesh.

**Figure 8:**
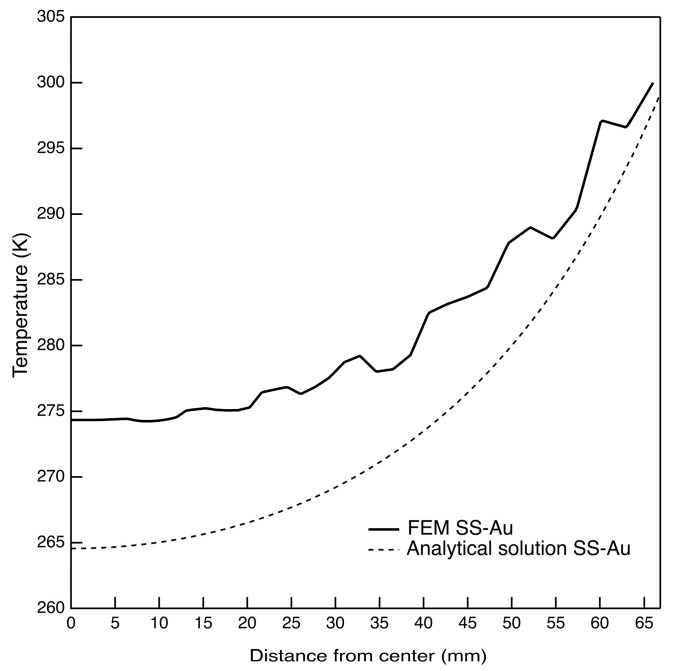
Radial temperature profiles of the simplified analytical solution vs. the detailed FEM.

In Figure 8, the FEM radial temperature is compared to that obtained by the analytical formulation. The FEM predicts 274 K at the filter center, while the analytical solution predicts 264 K with a ΔT radial gap of 26 K and 36 K, respectively. The temperature difference between the two solutions is then 10 K returning an 38% of error. This value is directly correlated with the approximations made by the analytical model (the absence of the cavity below the THF300 filter and the assumption of an equivalent material for the membrane, etc., …). In other words, it can be considered to be a measure of the level of approximation of the analytical model compared to the detailed FEM.

## 4. Results and Discussion

### 4.1. External Frame at 300 K

**Figure 9:**
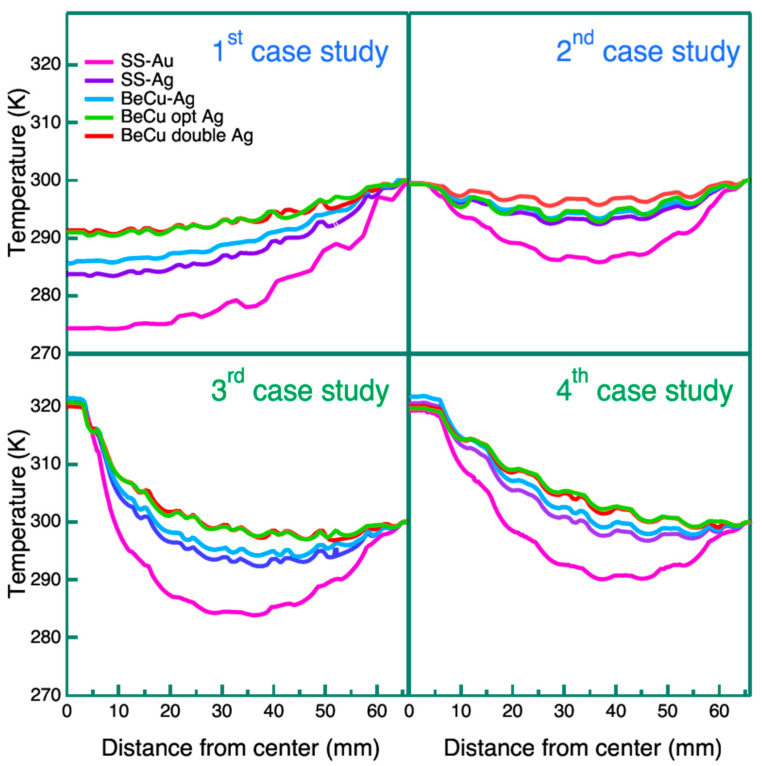
Radial temperature profiles for the first set of four case studies. Panels in the first row represent the passive conduction cases, while panels in the second row represent the active heating cases. The legend is the same for all graphs: model A SS-Au (magenta line), model B SS-Ag (purple line), model C BeCu-Ag (blue line), model D BeCu opt Ag (green line), and model E BeCu double Ag (red line).

The first case study investigates the influence of the mesh and plating materials on the radial temperature profile of the filter in passive conditions when the external frame temperature is fixed at 300 K. As expected, the poor thermal conductivity of the ultra-thin film causes a temperature drop inside each cell. Therefore, a wavy temperature radial profile is observed in all the curves of Figure 9 due to the hexagonal mesh pattern. This behavior is noticed for all the simulated temperature profiles in this work.

Looking at the top left panel of Figure 9, the effect of the plating on thermalization is clearly evident. Replacing the gold with the silver plating provides a significant boost in levelling the temperature though slightly increasing the BF. Comparing the curve of model A with model B, the temperature spread between the frame (300 K) and the center decreases from 25 K to 15 K.

The adoption of the BeCu 17,200 mesh material instead of the SS provides a modest thermalization improvement with respect to model B. The double mesh of model E leads to a further temperature rise in 7 K in the center (292 K) concerning model C, nominally maintaining the same BF. Model D, employing the BeCu 17,510 mesh and a 15 μm of Ag plating, provides almost the same outcomes as model E with double mesh, even if it improves the BF. A thickness of Ag larger than 15 μm, in addition to improving the thermal conductance, has negative effects on the mesh blocking factor and on the mechanical performances of the mesh under dynamic loads. For this reason, we considered 15 μm thickness as a maximum limit.

Looking at the top right panel of Figure 9, there are evident contributions of the Y-cross on thermalization levelling. In this case, as expected, the maximum drop in temperature is observed in the middle part of the radial profile, at about 35 mm from the center. At the filter center, the temperature is nearly the same as the external frame thanks to the high conductivity and cross-section of the Y-spokes. Model E, with double mesh, stands out as having better thermalization. As already shown in the first study case, the advantage of replacing gold with silver plating is clear with an increase of 7 K in the minimum temperature with respect to model A (Au plating). The temperature spread between the Ag plated models (B, C, D, and E) is less pronounced in this case, with all the curves showing a maximum ΔT gap of ~3 K. It is worth noting that the Y-cross solution, even if thermally advantageous, has a noticeable impact on the BF (>6%) and affects the image uniformity too.

In the third case, shown in the bottom left panel of Figure 9, a heat flux is applied to the central cell of the mesh which is assumed to be closed. Unlike the first two cases, here the active heating adds a parameter to the model thermal balance (the injected heat flux). To compare the five mesh combinations and plating, the heat flux value is set to reach 320 K at the central cell, and the area where the heat flux is injected is ~22 mm^2^. The values of the heat flux and of the deposited powers are reported in Table 4.

The bottom left panel of Figure 9 shows how the solution of injecting heat onto the central cell is not sufficient in levelling the temperature gradient, although the central hexagonal closed cell marginally influences the BF. In particular, the temperature of 320 K rapidly decreases with the distance from the center of the filter in all models. Comparing the five model configurations, again the Ag plating provides a better result than the Au with respect to thermalization. The temperature spread between the Ag plated models (B, C, D and E) is lower, with respect to the first case, with all the curves showing a maximum ΔT gap of ~4 K. Models D and E have similar behavior with reported lower temperatures of 299 K. It is interesting to note the trend of the deposited power listed in Table 4 where meshes with higher thermal conductivity (models D and E) require a higher heat flux to obtain the same central temperature (320 K), then resulting in better thermalization.

In the fourth case study, a distributed Joule heating is considered. The heat is generated by the Joule effect on the mesh material running a current from the mesh center (with the central cell being closed) through the outer frame. The latter is achieved by applying a voltage differential (ΔV). As in the previous third case, the active heating adds a parameter to the model thermal balance. Comparing the five mesh and plating configurations, the ΔV is set to reach 320 K at the central cell to have the same and comparable boundary conditions. The bottom right panel of Figure 9 shows how the Joule heating strategy provides slightly better performances with respect to the third case. The central temperature (320 K) rapidly decreases with the distance from the center of the filter for all five models. This outcome can be attributed to the mesh geometry that splits the current at each branch, gradually reducing the electrical resistance, and thus the Joule heat effect from center to perimeter. A significant contribution of the Ag plating is also reported for this case (models B, C, D and E), resulting in a better thermalization. Models D and E have similar behaviors, with a minimum temperature reported of 299 K. In Table 5, the values of the potential difference applied on the five models are reported.

### 4.2. Minimum Temperature of 320 K

In the passive condition cases (fifth and sixth), the thermal balance is obtained only through radiative and conductive heat fluxes. To meet the requirement of a 320 K minimum temperature throughout the filter, the external frame temperature is the only degree of freedom to be modulated.

**Figure 10:**
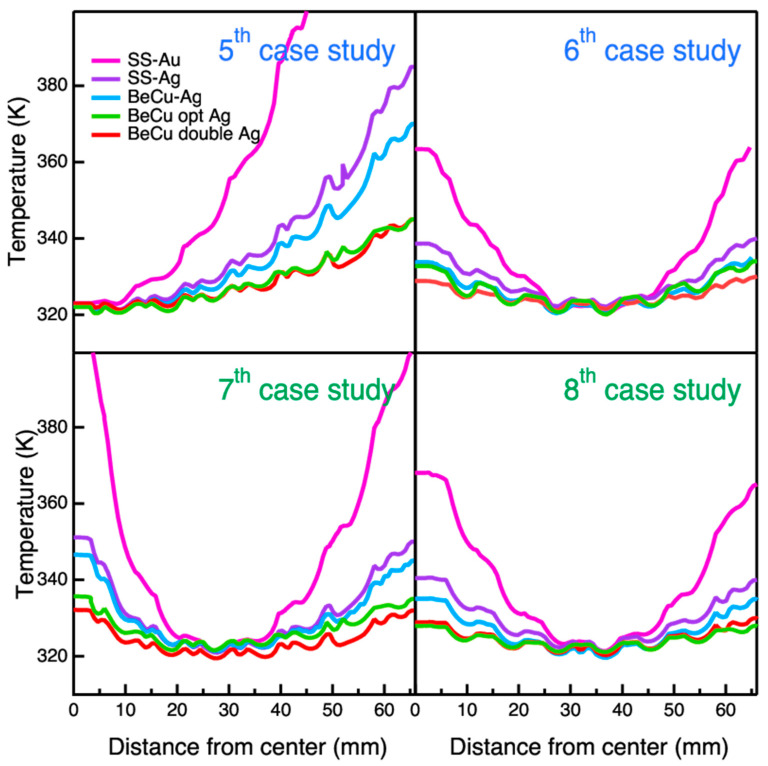
Radial temperature profile for the second set of four cases (from fifth to eighth) with the requirement of having at least 320 K throughout the entire filter. Panels in the first row represent the passive conduction cases, while panels in the second row the active heating cases. The legend is the same for all graphs: model A SS-Au (magenta line), model B SS-Ag (purple line), model C BeCu-Ag (blue line), model D BeCu opt Ag (green line), and model E BeCu double Ag (red line).

Figure 10 shows the radial temperature profiles for all models under the above requirement (T > 320 K).

In the top left panel of Figure 10, the temperature radial profile of model A is out of scale reaching a required temperature of ~600 K at the external frame to meet the requirement. The poor thermalization of model A is attributed to the inefficient thin gold plating. Comparing model A to model B, where the Ag replaces the Au plating, the temperature on the external frame to meet the requirement decreases down to ~200 K. Similar considerations of those reported in the first case can be stated here for models C, D and E. The best performances are reached by models D and E, with a needed external frame temperature of 345 K to meet the requirement.

As in the second case study, the effect of the aluminum Y-cross structure in the sixth case study is also evaluated. The top right panel of Figure 10 shows that the maximum temperature drop is observed in the middle of the radial profile (at about 35 mm from the center). By comparing model A to model B, the contribution of the Ag plating stands out with a ΔT of ~25 K. The Ag-plated models (B, C, D and E) behave similarly, with a ΔT of 10 K; model E, featuring the double mesh, has a slightly better performance.

As for the third case, a heat flux is injected in the seventh case on the central cell of the mesh which is closed on purpose. The injected heat flux, thus the deposited power, to meet the requirement is different with respect to the third case; the values are reported in Table 6. The bottom left panel of Figure 10 shows that the maximum drop in temperature is observed in the middle part of the radial profile (at about 30 mm from the center) since the heat flows both from the center (where the resistor is placed) and from the external frame.

As in the fifth case, the high temperature (of about 405 K) needed for model A to meet the requirement is evident. Comparing model A to model B, the Ag plating drops out the temperature of about 50 K at the external frame. The temperature spread between the Ag-plated models (B, C, D and E) is in the range of 20 K, with better performances achieved by models D and E.

In the eighth case, which is similar to the fourth, with the only exception of the additional degree of freedom of the external frame temperature, the heat is generated by the Joule effect on the mesh running a current from its center. The active heating and the external frame temperature are the two free parameters available to regulate the thermal balance of the system. These two parameters are balanced to minimize the temperature difference between the center and the perimeter of the filter, thus achieving the best leveling possible of the temperature with the minimal thermal load.

In Table 7, the values of the potential difference applied on the five models are reported.

As in the previously described seventh case study, the bottom right panel of Figure 10 shows the maximum drop in temperature in the middle of the radial profile but shifted on the frame side (~35 mm from the center) since the heat flows from both the center (where the main contribution of the joule heating is present) and from the external frame. Comparing the maximum temperature required at the frame to thermalize model A with Joule heating (368 K) and resistor heating (405 K), the Joule heating performs better.

As in all the studied cases, the Ag plating enhances the thermalization (ΔT ~28 K between model A and model B). The temperature difference between the Ag-plated models (B, C, D and E) is in the range of 13 K, with better performances achieved by models D and E. The thermal behavior of models D and E are very close, with a required temperature at the external frame of 330 K and 328 K, respectively.

## 5. Conclusions

The molecular contamination of the filters used on the beam path of X-ray detectors on space missions causes progressive loss of sensitivity at low energy during its operation. The outer filter, which is the first barrier for particles, should be kept at a temperature higher than the close environment to avoid it becoming a cold trap for molecular contamination. In the case of the Athena X-IFU instrument, whose environment is at 300 K, a requirement has been set to keep the outer thermal filter at a temperature higher than 320 K. This is the temperature value that we have targeted as the operational value in our simulations. In addition, it is still to be decided whether an even higher temperature is needed to run periodically in space a baking procedure for decontamination.

The current thermal filter design for the X-IFU instrument planned onboard the future Athena mission adopts Al coated polyimide thin film which has poor thermal conductance, and thus presents a temperature gradient between the outer and the inner part of the filter.

A parametric study on the thermalization of the outer thermal filter named THF300 has been carried out to retrieve the radial temperature profile of the filter using a static heat transfer steady state FEM. A total of eight case studies (the first to the eighth) with different boundary conditions have been considered. In addition, for each case, five different models (from A to E) with various meshes and plating materials combinations have been compared.

The parametric study proves that the baseline mesh material design (SS plated with 5 μm of Au) is not able to provide a uniform temperature throughout the filter. When gold is replaced with the equivalent mass of silver, the thermalization is enhanced by the higher thermal conductivity of Ag at 300 K (430 Wm^−1^ K^−1^) [30] concerning Au (317 Wm^−1^ K^−1^) [30]. The passive solutions with a heated frame (5th case) adopting an optimized design using the BeCu C17510 mesh plated with 15 μm of Ag (Model D) is the preferred solution, allowing to meet the requirement (T > 320 K throughout the filter) with a ΔT of ~25 K (~345 K at the external frame).

Other options that we have considered, such as the Y-cross solution and any active heating solutions, provide good thermalization, but they show some disadvantages. The Y-cross solution (sixth case), which is thermally advantageous, allows us to meet the requirement with a ΔT of only 10 K (~330 K at the external frame), but it adds 6% to the BF of the mesh (~3%) and introduces potential image non-uniformity at the focal plane. The active heating solutions (the seventh and eighth cases) introduce an additional risk, since a wire needs to be bonded to the central closed cell of the mesh and brought outside.

The adoption of a double mesh (model E) is shown not to produce significant improvements with respect to models D.

Based on the results of this analysis, we have identified a new baseline for the THF300 thermal filter of the Athena X-IFU with a mesh in BeCu alloy plated with 15 μm of silver. This choice allows us to implement a thermalization approach based only on passive conductive heating by the frame. The option of implementing active heating will also be considered if it will turn out to be necessary to perform periodically in space baking procedures for decontamination.

Demonstration model filter breadboards, according to the new design, have been manufactured proving technical feasibility. In addition, such breadboards have been tested with good results in an Ariane 6 vibration environment, providing useful hints for future developments. As an example we identified a potential advantage in launching the filters in a partial pressure more than in a vacuum to obtain some dumping of the filter maximum deformation.

The results of this analysis represent a very useful background, both in terms of results and methodology, for the new design of the X-IFU thermal filters stack in the reformulated Athena mission whose delta phase A has started in Q1 2024.

## Figures and Tables

**Table 1:** List of the 8 heating cases considered. From the 1st case to the 4th case, the temperature of the external frame is set to 300 K; from the 5th case to the 8th case the temperature throughout all the filter surface is set above 320 K. The light blue rows identify the cases based on the passive conduction approach, while the light green rows identify the studies based on the active heating approach.

Case Study	Description	Heating Strategy	Note
1st	Effects of mesh and plating material	Passive conduction	External frame at 300 K
2nd	Additional Y-cross over the mesh		
3rd	Heat flux injected on the filter center	Active heating	
4th	Joule heating by running a current		
5th	1st study + external frame heating	Passive conduction	Requirement to meet T > 320 K throughout the filter surface
6th	2nd study + external frame heating		
7th	3rd study + external frame heating	Active heating	
8th	4th study + external frame heating		

**Table 2:** Details about the mesh and plating material, plating thickness, mesh thickness and width, and the relative blocking factor for each studied model (SS: Stainless steel; BeCu: Beryllium copper alloy).

Model	Mesh Material (Single/Double)	Plating Material and Thickness [μm]	Mesh Bar Size h × w (h × w Plated) [μm]	Blocking Factor (%)
A—SS Au	SS AISI304 (single)	Au 5	80 × 40 (90 × 50)	2.37
B—SS Ag	SS AISI304 (single)	Ag 10	80 × 40 (100 × 60)	2.75
C—BeCu Ag	BeCu C17200 alloy (single)	Ag 10	80 × 40 (100 × 60)	2.75
D—BeCu opt Ag	BeCu C17510 alloy (single)	Ag 15	80 × 40 (110 × 70)	3.1
E—BeCu double Ag	BeCu C17200 alloy (double)	Ag 10	160 × 40 (200 × 60)	2.75

**Table 3:** Thermal conductivity of the adopted materials for the mesh, the plating, and the membrane components at the nominal temperature of 300 K.

Thermal Conductivity [W/m K] at 300 K [28]							
Al 5N	Gold 5N	AISI304 (SS)	Al oxide	Polyimide	Silver 5N	BeCu C17200	BeCu C17510
405	310	15.31	32	0.192	427	105	240

**Table 4:** Values of the deposited power and the heat flux for all the five models investigated in the third case.

Model	Heat Flux [mW/mm^2^]	Deposited Power [mW]
A SS-Au	0.8	17.6
B SS-Ag	1.5	33
C BeCu-Ag	1.7	37.4
D BeCu opt Ag	2.3	51
E BeCu double Ag	2.5	55

**Table 5:** Values of the voltage for all the five models investigated in the fourth case.

Model	Voltage [mV]	Dissipated Energy [mW]
A SS-Au	36	10
B SS-Ag	34	16
C BeCu-Ag	40	19
D BeCu opt Ag	22	26
E BeCu double Ag	25	26

**Table 6:** Values of the voltage for all the five models investigated in the seventh case.

Model	Heat Flux [mW/mm^2^]	Deposited Power [mW]
A SS-Au	2	44
B SS-Ag	1.8	39.6
C BeCu-Ag	1.8	39.6
D BeCu opt Ag	1.5	33
E BeCu double Ag	1.6	35.2

**Table 7:** Values of the voltage for all the five models investigated in the eighth case.

Model	Voltage [mV]	Dissipated Energy [mW]
A SS-Au	50	20
B SS-Ag	35	17
C BeCu-Ag	28	9
D BeCu opt Ag	22	14
E BeCu double Ag	19	15

## Data Availability

The data are not publicly available due to privacy.
